# Safety of embryo cryopreservation: insights from mid-term placental transcriptional changes

**DOI:** 10.1186/s12958-024-01241-7

**Published:** 2024-07-12

**Authors:** Qin-Yu Luo, Si-Wei Zhang, Hai-Yan Wu, Jia-Ying Mo, Jia-En Yu, Ren-Ke He, Zhao-Ying Jiang, Ke-Jing Zhu, Xue-Ying Liu, Zhong-Liang Lin, Jian-Zhong Sheng, Yu Zhang, Yan-Ting Wu, He-Feng Huang

**Affiliations:** 1 Key Laboratory of Reproductive Genetics (Ministry of Education), Department of Reproductive Endocrinology, Women’s Hospital, Zhejiang University School of Medicine, Hangzhou, China; 2 Obstetrics and Gynecology Hospital, Institute of Reproduction and Development, Fudan University, Shanghai, China; 3 International Institutes of Medicine, The Fourth Affiliated Hospital, Zhejiang University School of Medicine, Yiwu, China; 4 Research Units of Embryo Original Diseases, Chinese Academy of Medical Sciences, Shanghai, (No.2019RU056), China; 5 Shanghai Key Laboratory of Reproduction and Development, Shanghai, China

**Keywords:** Frozen embryo transfer, Embryo cryopreservation, RNA-seq, Placenta, Fetal growth, Imprinting gene

## Abstract

**Background:**

In recent years, with benefits from the continuous improvement of clinical technology and the advantage of fertility preservation, the application of embryo cryopreservation has been growing rapidly worldwide. However, amidst this growth, concerns about its safety persist. Numerous studies have highlighted the elevated risk of perinatal complications linked to frozen embryo transfer (FET), such as large for gestational age (LGA) and hypertensive disorders during pregnancy. Thus, it is imperative to explore the potential risk of embryo cryopreservation and its related mechanisms.

**Methods:**

Given the strict ethical constraints on clinical samples, we employed mouse models in this study. Three experimental groups were established: the naturally conceived (NC) group, the fresh embryo transfer (Fresh-ET) group, and the FET group. Blastocyst formation rates and implantation rates were calculated post-embryo cryopreservation. The impact of FET on fetal growth was evaluated upon fetal and placental weight. Placental RNA-seq was conducted, encompassing comprehensive analyses of various comparisons (Fresh-ET vs. NC, FET vs. NC, and FET vs. Fresh-ET).

**Results:**

Reduced rates of blastocyst formation and implantation were observed post-embryo cryopreservation. Fresh-ET resulted in a significant decrease in fetal weight compared to NC group, whereas FET reversed this decline. RNA-seq analysis indicated that the majority of the expression changes in FET were inherited from Fresh-ET, and alterations solely attributed to embryo cryopreservation were moderate. Unexpectedly, certain genes that showed alterations in Fresh-ET tended to be restored in FET. Further analysis suggested that this regression may underlie the improvement of fetal growth restriction in FET. The expression of imprinted genes was disrupted in both FET and Fresh-ET groups.

**Conclusion:**

Based on our experimental data on mouse models, the impact of embryo cryopreservation is less pronounced than other in vitro manipulations in Fresh-ET. However, the impairment of the embryonic developmental potential and the gene alterations in placenta still suggested it to be a risky operation.

**Supplementary Information:**

The online version contains supplementary material available at 10.1186/s12958-024-01241-7.

## Background

In 1984, the world’s first FET baby was produced at the Queen Victoria Medical Centre in Melbourne, Australia. In 1990, vitrification was first successfully applied in clinical practice [1]. Since then, FET has been widely promoted and applied worldwide. Disturbingly, however, the safety of this technique is still up for debate. Many studies have reported an elevated risk of LGA babies born from FET [2–4]. A recent large cohort study from Nordic countries showed that mothers conceiving FET babies were suffering with a greater chance of hypertensive-related disease in pregnancy [5]. Furthermore, some studies focusing on long-term impact reported that FET children may be more prone to some specific types of cancers, such as leukemia and tumors of the sympathetic nervous system [6, 7]. However, it should be noted that conflicting findings have been reported by some other studies. For instance, a meta-analysis reported that FET pregnancies had a lower risk of preterm delivery and lower birthweight compared to Fresh-ET [4]. In addition, a retrospective study involving 110 Fresh-ET and 136 FET pregnancies suggested that FET led to higher pregnancy rates [8]. These contrasting results, along with the significant benefits of FET for fertility preservation, have contributed to the growing endorsement and promotion of FET in clinics. Thus, it is imperative to explore the full spectrum of influences of FET on both mothers and babies.

The placenta serves as the primary regulator of intrauterine fetal growth, playing a crucial role in nutrient exchange between mothers and babies. The normal development and proper functioning of the placenta are essential for ensuring the well-being of both mothers and babies and compromised placental function underlies a range of perinatal complications. Extensive research has indicated that insufficient nutrient transportation by the placenta was one of the major causes of intrauterine growth restriction [9]. Moreover, the defect in spiral artery remodeling of the placenta is widely taken as an important pathogenic mechanism of pre-eclampsia [10]. Considering the increasing number of studies that report elevated risks of placenta-associated perinatal complications following FET [5, 11, 12], adverse effects of FET on placental development are strongly suggested. Furthermore, many recent studies have found that abnormal intrauterine conditions and impaired fetal growth contribute to long-term health consequences of offsprings through intrauterine fetal programming [13]. Taken together, we believe the alterations of the placenta in FET babies are an ideal medium reflecting the impact of FET on fetal development, and they can also provide hints of long-term effects posed by FET.

Imprinted genes make up only a small proportion of all genes. However, they attract considerable attention in ART-related articles. The first imprinted gene, *Igf2r*, was discovered by Denise Barlow in 1991. So far, over 150 imprinted genes have been reported in humans and mice [14], and most of these genes are actively expressed in placentae and play a pivotal role in the development of placentae and fetuses [15]. Some studies have reported alterations in imprinted genes in fetuses and placentae following ART procedures, including Fresh-ET and FET [16–18]. Also, researchers have demonstrated a higher incidence of imprinting-related diseases, such as Beckwith-Wiedemann syndrome, among ART-conceived children compared to those conceived naturally [19, 20]. Therefore, we also paid special attention to the expression of imprinted genes in the present study.

This study aimed to assess the safety of embryo cryopreservation within the framework of the entire assisted reproductive strategy. By comparing differentially expressed genes (DEGs) from various group comparisons (Fresh-ET vs. NC, FET vs. NC and FET vs. Fresh-ET), we dissected the effects specifically attributed to embryo cryopreservation and evaluated the extent of influence exerted by FET compared to Fresh-ET. Furthermore, we explored the alterations of imprinted genes induced by FET as well as Fresh-ET.

## Methods

### Experimental design

**Fig. 1:**
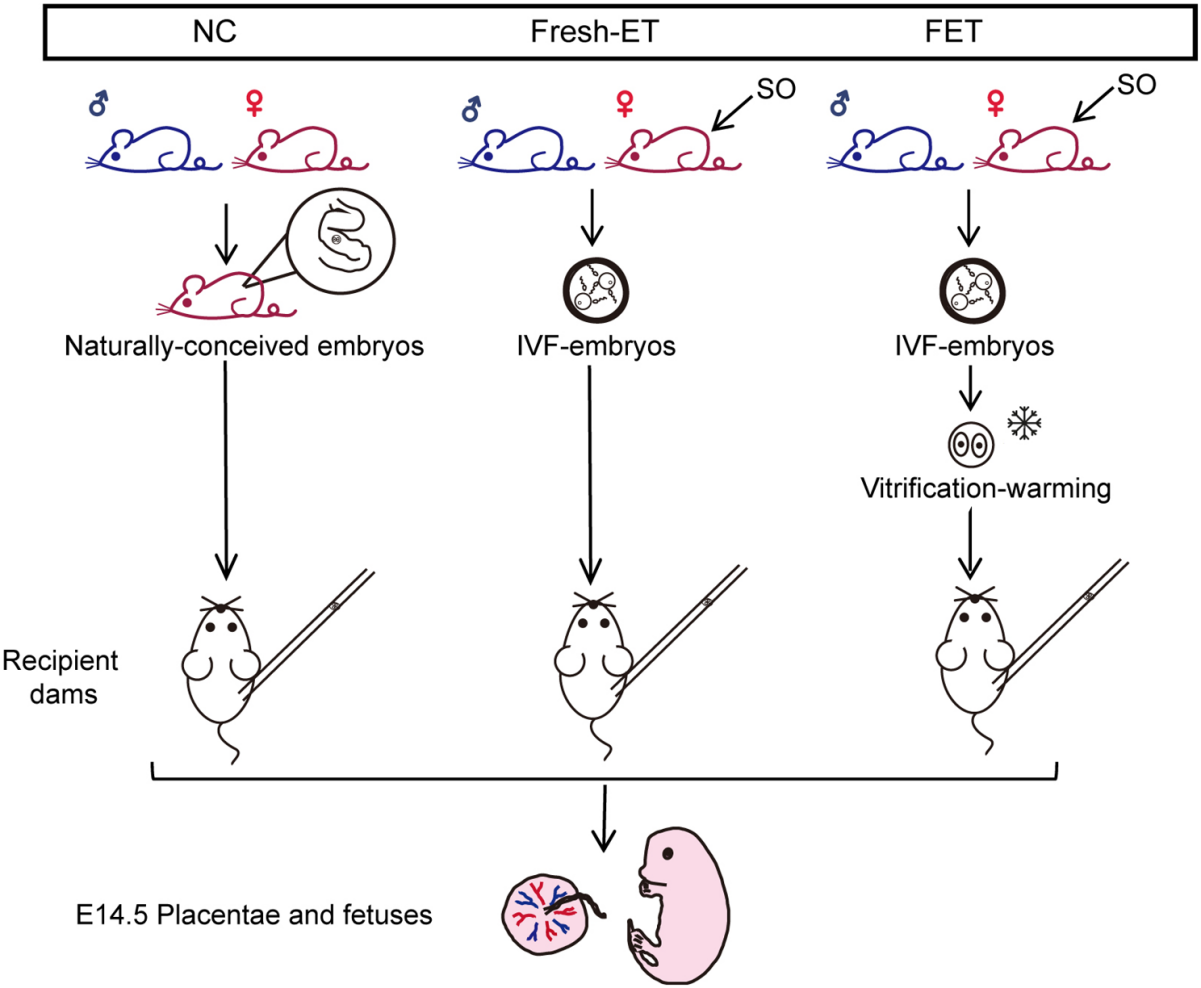
Experimental design. Three experimental groups were set up here: NC group, Fresh-ET group and FET group. In NC group, mice were mated naturally, and 2-cell embryos were obtained from the oviducts of female mice on E1.5. In Fresh-ET and FET groups, embryos were obtained by IVF. Additionally, the embryos in FET group underwent vitrification and thawing at the 2-cell stage. All three groups had their embryos transferred to pseudo-pregnant dams on E1.5, and tissue collection took place on E14.5. SO, superovulation

Animal rearing conditions and experimental procedures were carried out in strict accordance with the requirements of the Animal Care and Use Committee, School of Medicine, Zhejiang University. Three experimental groups were set up in this article, the NC group, the Fresh-ET group and the FET group. In NC group, C57BL/6J mice were mated naturally, and the presence of virginal plugs in female mice was confirmed the following morning. For Fresh-ET and FET groups, sperms and oocytes were collected from C57BL/6J mice for in vitro fertilization (IVF). Vitrification and warming were conducted on FET group at 2-cell stage. All C57BL/6J male mice used in experiments were 12–16 weeks old, all C57BL/6J female mice were 6–8 weeks old. Embryos of three groups were all collected at 2-cell stage and transferred into pseudo-pregnant ICR mice. The day of embryo transfer was designated as E1.5, and tissue collection and weight measurements were conducted on E14.5 (Fig. 1).

### IVF and in vitro culture (IVC)

All oocyte-donor mice in Fresh-ET group and FET groups received intraperitoneal injection of PMSG (5IU; Sudgen, China) and HCG (10IU; Sudgen, China) before oocyte retrieval. The interval between the two injections was controlled at 46–48 h. Approximately 14–16 h after HCG injection, female mice were sacrificed, and the cumulus-oocyte complexes were retrieved from bilateral ampullae and placed in pre-prepared droplets of HTF (Sudgen, China). Sperm was collected from bilateral epididymides of male mice. Fresh semen was placed in a droplet of CYTH (Sudgen, China) for half an hour and then transferred to HTF. Sperm and oocytes in HTF can get fertilized autonomously. Zygotes were washed and observed 24–28 h post-fertilization. Some of the zygotes were transferred to pseudo-pregnant ICR mice, while the remaining zygotes were incubated in vitro for another 72 h until they reached the blastocyst stage.

### Embryo vitrification and warming

Vitrification was performed at 24–28 h post-IVF. Only embryos that had reached 2-cell stage were selected for vitrification, while unfertilized or delayed embryos were discarded. Typically, at this time point, more than 90–95% of the eggs had been fertilized and developed into the 2-cell stage. Occasionally, there would be a batch with an abnormally lower rate of 2-cell embryos, and these batches would not be used. EFS40 and EFS20 were used for vitrification in accordance to the protocols provided by the supplier (Sudgen, China). Briefly, embryos were first placed in EFS20 droplets at room temperature for 2 min and then immediately transferred to EFS40 droplets in a cryotube for 1 min at 0 ◦C. Subsequently, cryotubes were placed into liquid nitrogen and stored for one week.

For warming, the cryotubes were opened and exposed to room temperature for 30s once detached from liquid nitrogen. Then, 0.75Su was added to cryotubes to dissolve EFS40 and the embryos within. We gently mixed the solutions contained in cryotubes and then transferred them to a 10 mm petri dish (Corning, United States), in which recovered 2-cell embryos were collected under a microscope. These embryos were then transferred to 0.25Su for further recovery. Finally, well-formed 2-cell embryos were transferred to KSOM (Sigma, China). For embryos intended for in vitro experiments, their status was checked one hour later, and any dead embryos were removed while the remaining embryos were placed back in the incubator for further culture. Normally, only a few embryos were found to be dead at this stage. For embryos planned for transfer to recipient dams, they were cultured for about an hour post-thawing before transplantation.

### Embryo collection and transfer

ICR surrogate female mice were obtained by mating with ICR vasectomized male mice. Successful copulation was confirmed by the presence of a vaginal plug the next day morning, which was calculated as E0.5. All embryos were transferred at 2 cell stage on E1.5 into pseudo-pregnant recipient mice. For NC group, embryos were flushed out from the oviducts of C57 mothers and transferred to ICR dams immediately. For Fresh-ET group, embryos were collected from in vitro culture medium 28 h post IVF. Considering the limitation of experimental conditions, we did not know the exact time when the oocyte and sperm were fertilized, so here the time of IVF refers to the moment sperm suspension was added to HTF droplets. For FET groups, we transferred the embryos about an hour post-warming to avoid dead embryos. Recipient mice were anesthetized and prepared before we dealt with the embryos so to minimize the time embryos were exposed to the external environment. Each side of the oviduct received 8–12 embryos, the operational details of embryo transfer were in accordance to a Laboratory Manual from Nagy A. et al [21].

### Placenta dissection and RNA preparation

For all three groups, fetuses and placentae were harvested on E14.5. Pregnant mice were euthanized by cervical dislocation after being anesthetized, and the bilateral uterus was quickly removed from the postmortem mice. The weight of placentae and fetuses were recorded after disscection. In detail, we first opened the pelvic cavity of pregnant mice, exposing the uterus containing fetuses fully in view. Next, the connection between the uterus and the dorsal peritoneum was cut so that the entire uterus could be completely removed. The removed uterus was transferred to a clean petri dish. Using two forceps, we clamped the uterus at the back of fetal mice and pulled in reverse to tear the myometrium and the amniotic membrane. The fetus, along with amniotic fluid, came out naturally. After cutting the umbilical cord, we cleaned the fetal mouse with gauze and transferred it to another new dish. Separating the entire placenta from the uterus involved holding the cord’s end with one forceps and the uterine wall with another. It was usually easy to get a full placenta. Maternal decidua should be removed as much as possible in this step. Following a quick wipe-down, the placenta was positioned beside its fetus in the dish. The entire process was done swiftly and skillfully. After recording the weight, placentae were washed in PBS and preserved at -80℃ for later experiments. RNA extraction was performed by RNAfast200 kit according to the manufacturer’s instructions (Fasta, Shanghai, China). In total, we collected 22 samples from the NC group, 31 samples from the Fresh-ET group, 41 samples from the FET group. RNA concentration and quality were analyzed by NanoDrop (Thermo Fisher, USA).

### RNA-seq and data analysis

RNA samples from NC, Fresh-ET and FET groups were pooled before sequencing. The details for each group’s pools were as follows: (1) NC group, 2 replicates, a total of 20 RNA samples in 2 pools; (2) Fresh -ET group, 2 replicates, a total of 31 RNA samples in 2 pools; (3) FET group, 2 replicates, a total of 41 RNA samples in 2 pools. The concentration and quality of RNA samples were checked by Agilent 2200 Tape Station (Agilent, United States). Libraries were constructed by Stranded mRNA-seq Kit (KAPA) according to the manufacturer’s instructions. The sequencing of libraries was performed on DNBseq platform. The raw sequencing data underwent filtering by SOAPnuke (v1.4.0) [22] to obtain clean reads. Filtered data were then aligned to the reference genome sequence (GCF_000001635.27_GRCm39) using Bowtie2(v2.2.5) [23]. Differential expression analysis of the gene count matrix was conducted by DEseq2 [24]. Genes with P value < 0.05 and |fold change| ≥ 1.5 were considered as differentially expressed genes (DEGs). Gene ontology (GO) enrichment analyses and kyoto encyclopedia of genes and genomes (KEGG) pathway analyses for DEGs were performed by DAVID 2021 website [25].

### RT-QPCR

Based on the analysis of the sequencing data, we selected some genes to validate their expression levels, including *Cxcl14*, *Klk4*, *Utf1*, *cd209c*, *Ndufb2*, *Atp5e*, *Ndufa2*, and *Ndufc1*. RNA samples were reversely transcribed into cDNA by PrimeScript™ RT reagent Kit (Takara, Japan). TB green Premix EX Taq kit (Takara, Japan) was used for quantitative real-time PCR (RT-QPCR). The Roche LightCycler® 480 System (Roche, Switzerland) was used to run the PCR reactions. Relative concentration of each sample was calculated according to CT values and the results were then analyzed by GraphPad Prism6. Student’s t-test and Chi-square test were used to compare data from groups, and *p* < 0.05 was taken to be statistically different. In all experiments, we consistently used GAPDH as the internal reference gene.

## Results

### Embryo cryopreservation led to decreased embryonic developmental potential

**Fig. 2:**
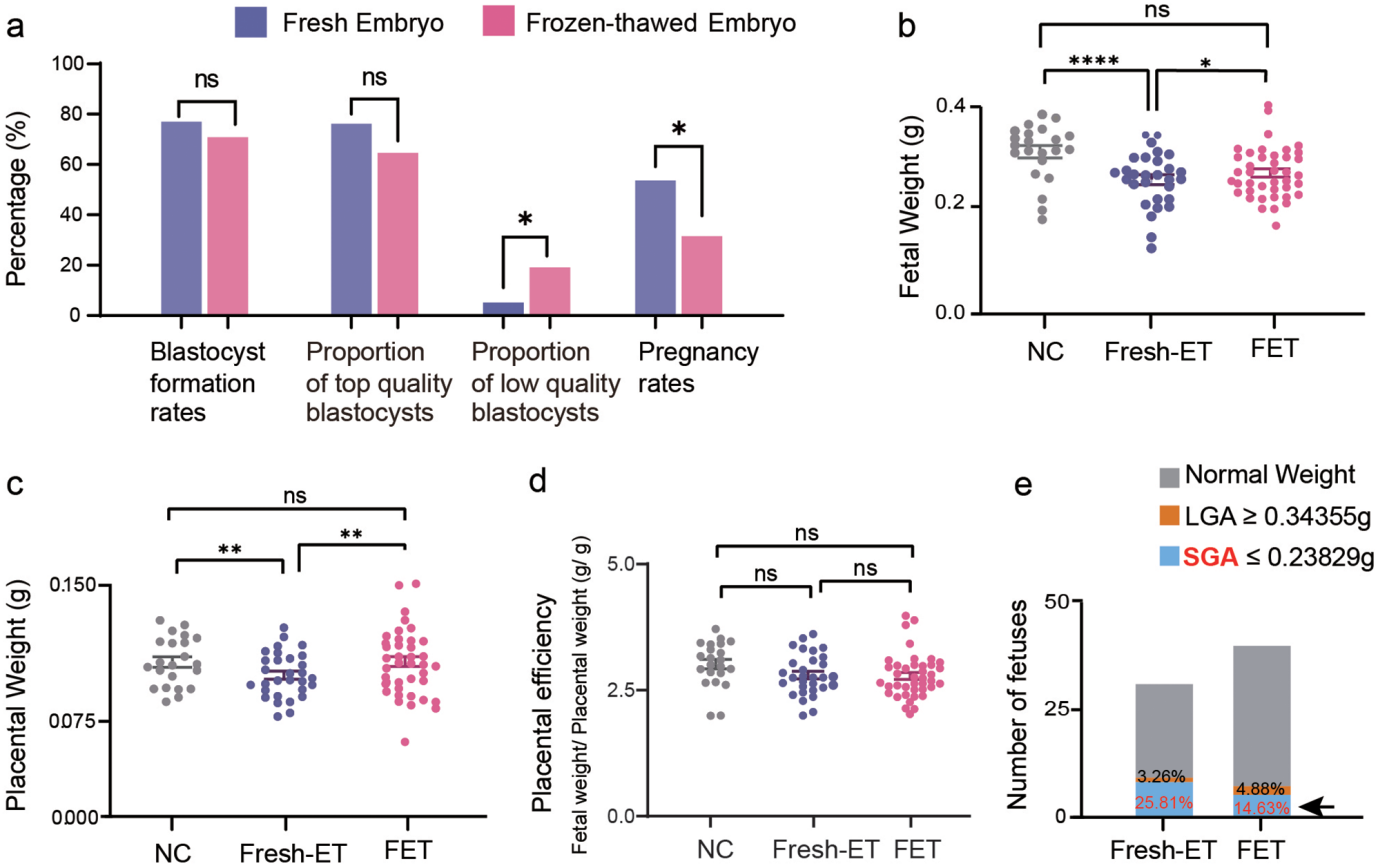
The evaluation of embryonic developmental potential and fetal growth (**a**) The rate of blastocyst formation and the proportion of top-quality blastocysts of frozen-thawed embryos showed no significant difference(*p* = 0.7127, *p* = 0.1047). The proportion of low-quality blastocysts wasd significantlyu increased (*p* = 0.029). FET group exhibited significantly lower pregnancy rates compared to Fresh-ET group (*p* = 0.006997) and NC group (*p* = 0.01201). Pregnancy rates, the ratio of the number of fetuses obtained on E14.5 to the number of all embryos transfer on E1.5. (**b**) Fetal weight in NC, Fresh-ET and FET groups. (**c**) Placental weight in NC, Fresh-ET and FET groups. (**d**) Placental efficiency in NC, Fresh-ET and FET groups. Placental efficiency, the value of the fetal weight divided by placental weight. (**e**) Incidence rates of SGA and LGA in Fresh-ET and FET groups. No significant difference. (*p* = 0.06968, 0.8085) The number of samples: 22 placentae and fetuses of NC group; 31 placentae and fetuses of Fresh-ET group; 41 placentae and fetuses of FET group. LGA, large for gestational age; SGA, small for gestational age. *p < 0.05; **p < 0.01; ****p < 0.0001

In this study, we established three experimental groups to evaluate the effects of embryo cryopreservation on the development of fetuses and placentae (Fig. 1). To determine the impact of embryo cryopreservation on early embryos before implantation, we cultured embryos in vitro and calculated their blastocyst formation rates. Notably, in vitro experimental results revealed a lower rate of blastocyst formation following embryo cryopreservation, although there was no statistically significant difference (*p* = 0.7127, Table 1; Fig. 2a). We further graded all blastocysts according to the criteria from Cheng, T.-C. et al [26]. Results showed that frozen embryos had a lower proportion of top-quality blastocysts and a significantly higher proportion of low-quality blastocysts compared to fresh embryos (*p* = 0.1047, *p* = 0.029; Table 1; Fig. 2a). These findings indicated impaired developmental potential of frozen embryos before implantation, aligning with previous animal studies [27]. For embryos transferred back in vivo, we calculated the pregnancy rates for all three groups. We found a decrease in pregnancy rates in the Fresh-ET group compared to the NC group, although this difference was not statistically significant (*p* = 0.6789). For the FET group, the pregnancy rate exhibited a striking decline compared to the other two groups (*p* = 0.01201, *p* = 0.006997; Fig. 2a). The unmatched decrease in blastocyst formation and pregnancy rates in FET suggested that post-implantation embryonic development was also damaged. It is worth noting that this finding is inconsistent with some clinical research. A multi-center, randomized trial published in 2018 reported a comparable live birth rate between FET and Fresh-ET, and other studies even supported an elevated pregnancy rate in FET compared to Fresh-ET [8, 28]. We speculate that this discrepancy might be attributed to the different hormone cycles employed in Fresh-ET and FET treatments in clinics. Numerous studies have suggested that high levels of stimulating hormones during artificial cycles could negatively impact pregnancy rates in Fresh-ET [29]. In our experiments, we adopted natural cycles in both groups to eliminate the unwanted interference on the final results. Hence, the gap between animal models and clinical practice may lead to the divergent manifestation of pregnancy rates between humans and mice. To sum up, we observed a decreased developmental potential of frozen embryos both before and after implantation.

**Table 1:** Counting and grading of blastocysts 96 h after IVF

	Number of total embryos	Blastocyst formation rates	Grading of blastocysts	
Fresh Embryos	126	76.98% (97/ 126)	Top quality	74/ 97 (76.28%)
			Intermediate quality	17/ 97 (17.52%)
			Low quality	5/ 97 (5.15%)
Frozen- thawed Embroys	96	70.83% (68/ 96)	Top quality	44/ 68 (64.70%)
			Intermediate quality	11/ 68 (16.18%)
			Low quality	13/ 68 (19.12%)

### FET retraced the restrictive effects of intrauterine fetal growth led by Fresh-ET and showed comparable fetal weight to NC group

To evaluate the impact of embryo cryopreservation on intrauterine development of fetuses and placentae, we collected the plancentae and fetuses on E14.5 and measured their weight. Both fetal and placental weights were significantly reduced in Fresh-ET group compared to NC group (*p* < 0.0001, *p* = 0.0024; Fig. 2b-c). Interestingly, FET group showed significantly increased weights of both placentae and fetuses compared to Fresh-ET group (*p* = 0.0295, *p* = 0.00207; Fig. 2b-c). Thus, no significant difference was observed between FET and NC groups. There was no difference in placental efficiency among all groups (Fig. 2d). In addition, we took the fetal weight from NC group as a reference and defined large for gestational age (LGA) and small for gestational age (SGA) as weight below the 10th percentile of the mean weight and above the 90th percentile of the mean weight, respectively. The proportion of LGA and SGA fetuses in Fresh-ET and FET groups was calculated (Fig. 2e). The rate of SGA was as high as 25.18% in Fresh-ET group. However, it dropped to 12.5% in FET group. Meanwhile, the rate of LGA was only 3.2% in Fresh-ET group and 5% in FET group. No statistical significance was reached for either SGA or LGA between groups; this may be due to the relatively small size of samples. In summary, we observed a significant decrease in fetal weight in Fresh-ET compared to NC group, whereas the decline was somehow reserved in FET.

### A significant number of Fresh-ET altered genes persisted with their changes in FET

**Fig. 3:**
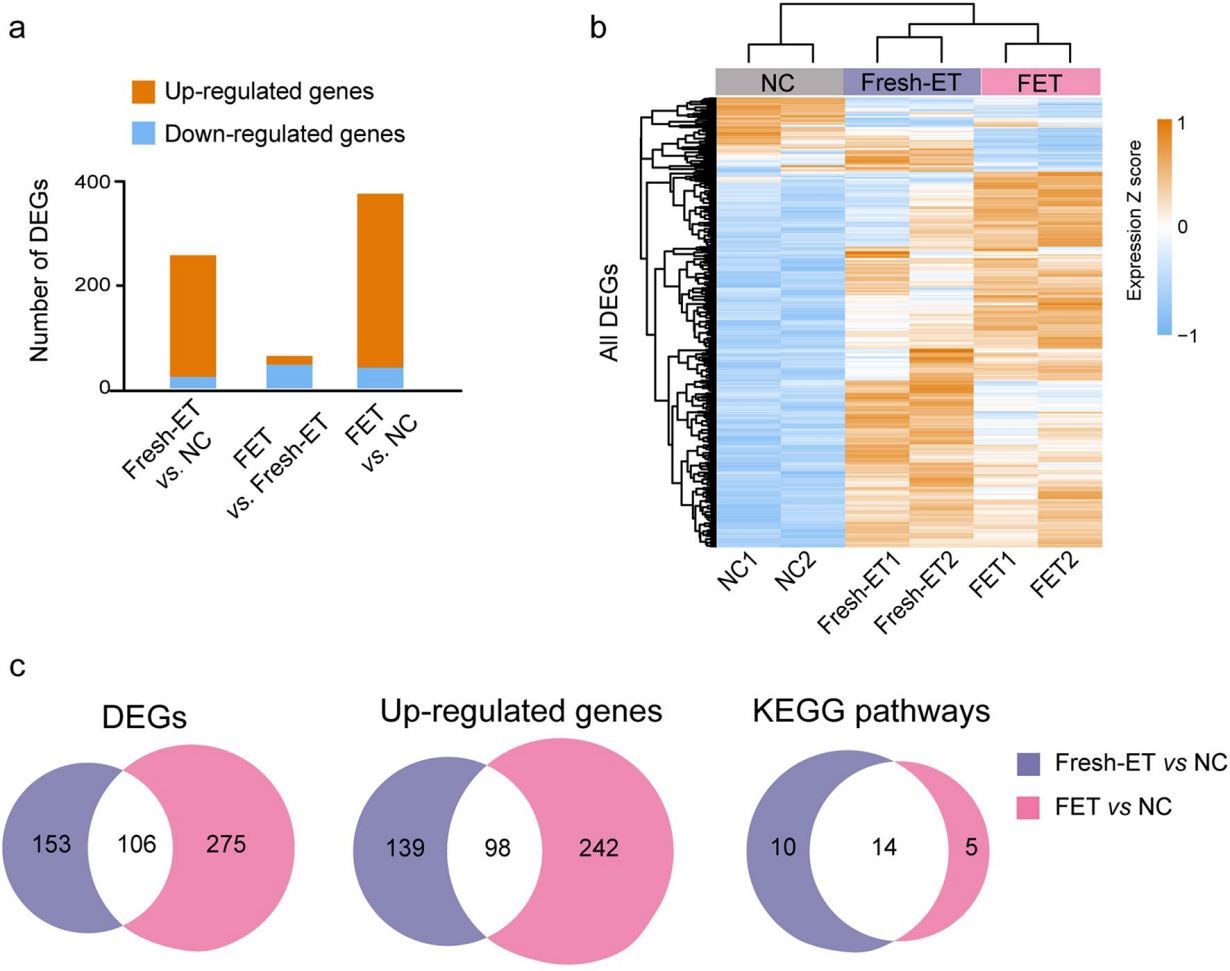
A significant number of Fresh-ET altered genes continued their changes in FET. (**a**) The number of DEGs from different comparisons, Fresh-ET vs. NC, FET vs. Fresh-ET, and FET vs. NC (*n* = 259, 63, 381). Orange columns represent the up-regulated genes (*n* = 237, 17, 340), and the blue columns represent the down-regulated genes (*n* = 22, 46, 41). (**b**) The unsupervised hierarchical clustering heat map of all DEGs, including Fresh-ET vs. NC, FET vs. Fresh-ET, and FET vs. NC. Each column represents data from one sample and each row shows the expression level of this gene across three groups. (**c**) The number of the overlapped DEGs, up-regulated genes and KEGG pathways of DEGs between Fresh-ET vs. NC and FET vs. NC

**Fig. 4:**
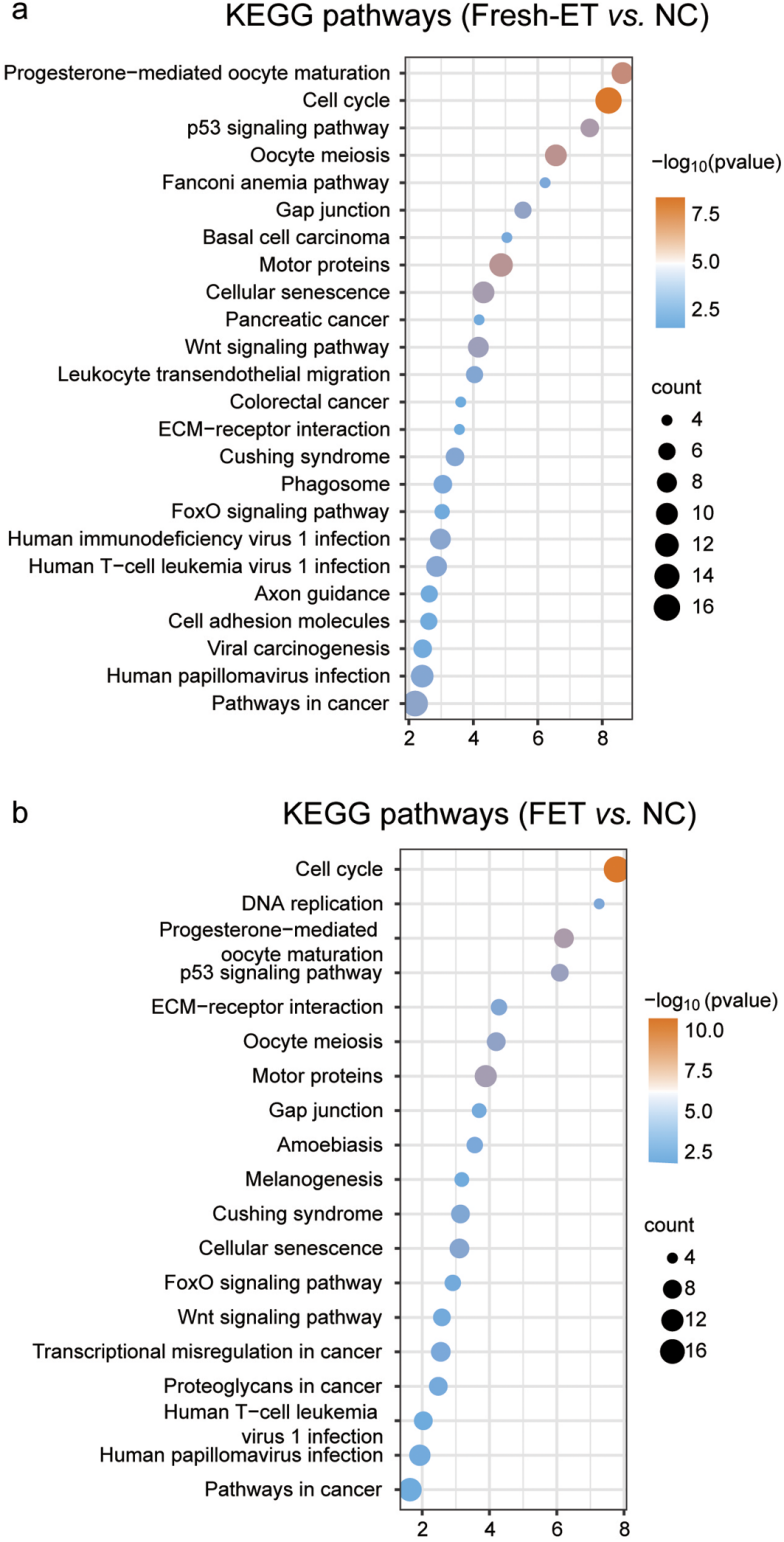
KEGG analysis of DEGs. (**a**) KEGG pathway analysis of DEGs of Fresh-ET vs. NC. (**b**) KEGG pathway analysis of DEGs of FET vs. NC.

The placenta serves as the primary regulator of intrauterine fetal growth, playing a crucial role in nutrient exchange between mothers and babies. The dysfunction of the placenta can lead to impaired developmental potential of the fetus, resulting in abnormal manifestations, such as SGA or low birth weight. The formation and growth of the mouse placenta primarily take place before the end of mid-term gestation (E9.5-E14.5). Furthermore, the weight and volume of the mouse placenta do not change much in late-term gestation (E14.5-E18.5) [30, 31]. Hence, we opted to examine the growth and development of the placentae and fetuses on E14.5. RNA-seq was performed on E14.5 placentae across three groups. Differentially expressed genes (DEGs) were calculated based on the threshold of |fold change|>1.5 and p value < 0.05. Results showed that there was a total of 259 DEGs for Fresh-ET vs. NC, including 22 down-regulated genes and 237 up-regulated genes. For FET vs. NC, 381 DEGs were identified, including 41 down-regulated genes and 340 up-regulated genes. In addition, 63 DEGs were detected between Fresh-ET and FET, with 17 genes getting up-regulated and 46 genes getting down-regulated in the FET group compared to the Fresh-ET group (Fig. 3a). All DEGs are presented in hierarchical cluster analysis in Fig. 3b, and the gene list is provided in additional file (**see Additional file 1**). Considering the number of DEGs, the effects on gene expression alterations owing to embryo cryopreservation were seemingly less pronounced compared to other manipulations involved in Fresh-ET, including super-ovulation and in vitro fertilization. Moreover, we noticed a considerable overlap of the DEGs lists and their corresponding pathways between Fresh-ET vs. NC and FET vs. NC (Fig. 3c). One hundred and six genes were shared between the two DEGs lists, among which 98 were up-regulated in both groups. KEGG pathway analysis revealed that DEGs of Fresh-ET vs. NC and FET vs. NC comparisons were both related to cell cycle, p53 signaling pathway, cellular senescence, FoxO signaling pathways and Wnt signaling pathway (Fig. 4a-b). Previous studies have reported that the p53 signaling pathway, FoxO signaling pathways, cellular senescence, and Wnt signaling pathway play a crucial role in the development of placentae and fetuses. Deficiencies of these functions contribute to perinatal complications, such as stillbirth, miscarriage, pre-eclampsia, and impaired fetal growth [32–36]. In our study, we noticed an elevated expression of *Wnt5a*, which is associated with intrauterine fetal growth restriction in a clinical study involving 52 samples from singleton pregnancies [37]. Another protein involved in the Wnt signaling pathway, *Sfrp5*, was also found to be up-regulated in both Fresh-ET and FET groups in our study. It is worth noting that the concentration level of *Sfrp5* has been reported to be elevated in the serum of pre-eclampsia patients [38]. Except for the overlapped pathways, DEGs of FET vs. NC were specifically involved in amebiasis, melanogenesis, and DNA replication (Fig. 4b). Based on the comparison of DEGs and their corresponding pathways, we concluded that FET inherited a significant portion of alterations triggered by Fresh-ET.

### Unexpected gene expression reversal in FET placenta

**Fig. 5:**
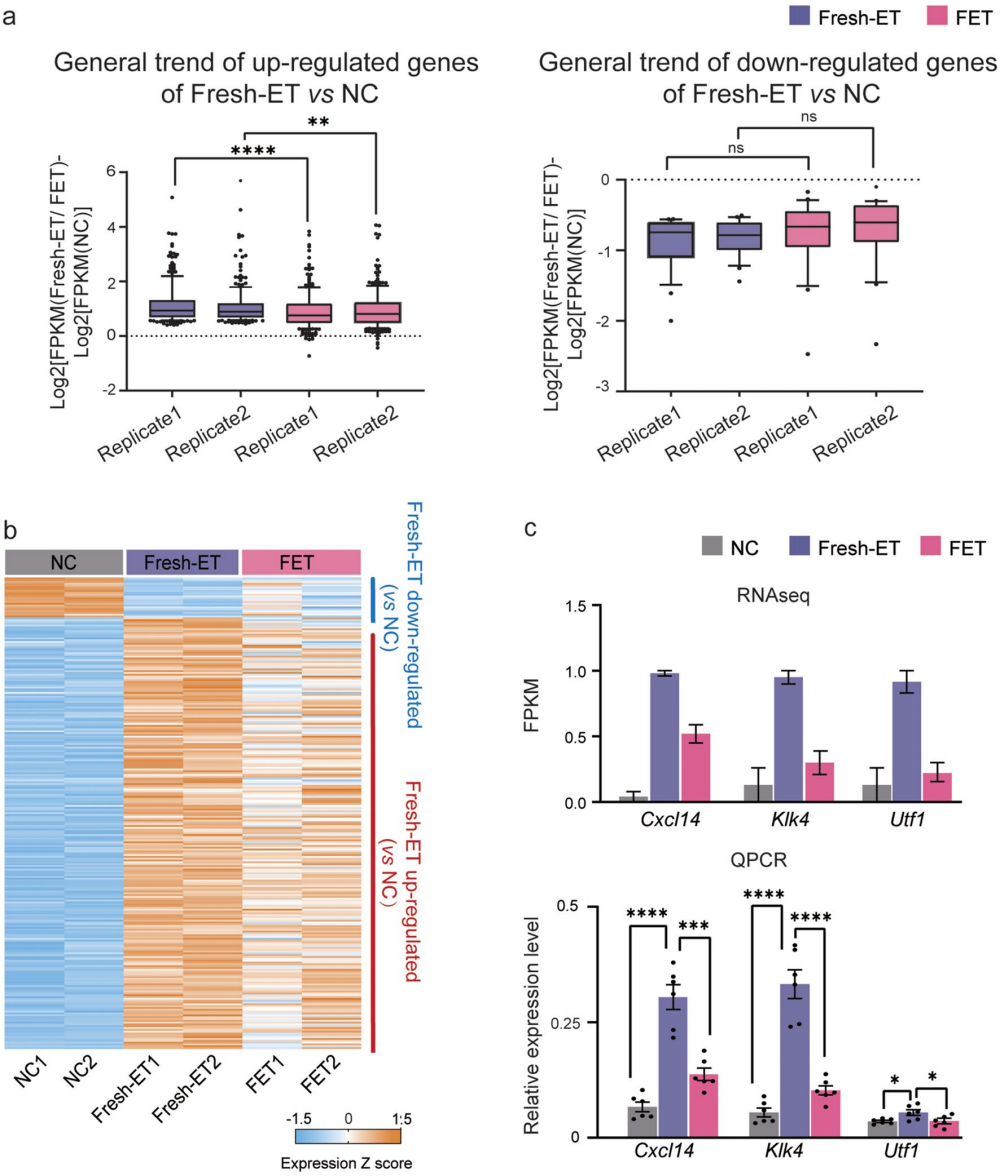
Unexpected gene reversal in FET placenta. (**a**) Left: The general changing trend of up-regulated genes (Fresh-ET vs. NC) in Fresh-ET and FET groups. The boxplots represented the expression difference of the genes with respect to NC in Fresh-ET and FET groups, calculated as Log_2_[FPKM (Fresh-ET/ FET)- Log_2_[FPKM(NC)]. The replicate 1 refers to Log_2_[FPKM (Fresh-ET 1/ FET1)- Log_2_[FPKM (NC1)]; The replicate 2 refers to Log2[FPKM (Fresh-ET2/ FET2)- Log2[FPKM (NC2)]. Non-parametric test detected statistical significance between each pair of replicates. Right: The general changing trend of down-regulated genes (Fresh-ET vs. NC) in Fresh-ET and FET groups. The expression and calculation methods involved are the same as the left one. No statistical significance was detected. (**b**) Heatmap of DEGs from Fresh-ET vs. NC. Each column represents data from one sample and each row shows the expression level of this gene across three groups. (**c**) The expression level of three representative genes that were restored in FET (*Cxcl14*, *Klk4*, and *Utf1*). Data came from RNA-seq (upper graph) and QPCR (lower graph). *P < 0.05, ***P < 0.01,***P < 0.001, ****p < 0.0001

Our previous analysis found that FET vs. NC inherited a considerable part of the changes in Fresh-ET vs. NC. However, it is noteworthy that a significant portion of genes altered in Fresh-ET somehow showed no significant change in FET. To better understand the divergent gene alteration spectrums of Fresh-ET and FET, we scrutinized the expression of all DEGs of Fresh-ET vs. NC in FET. Figure 5a demonstrated the general changing trend of these DEGs (Fresh-ET vs. NC) in Fresh-ET and FET groups, divided and presented as up-regulated genes and down-regulated genes, respectively. The boxplots in these two graphs represented the expression difference of the relating DEGs with respect to NC in Fresh-ET and FET groups. In the graph depicting the up-regulated genes, it is evident that the expression difference of these genes with respect to NC in FET group converged to zero. This implied that these genes that were up-regulated in Fresh-ET had a tendency to fall back to the expression level of the NC group in FET. This trend was observed on both replicates, and the non-parametric test showed that the extent of this trend has reached statistical significance. Similarly, for the genes underwent down-regulated expression in Fresh-ET, we also found a change with a smaller difference in the FET group. Whereas statistical tests did not detect a significant tendency for these genes to be regulated back in FET. Based on these findings, we propose that rather than strictly inheriting the gene alterations from Fresh-ET, FET surprisingly manifested a regression trend on some genes altered in Fresh-ET. In Fig. 5b, the expression levels of all DEGs of Fresh-ET vs. NC were shown in three groups, of which the most significant reserved genes, *Cxcl14, Klk4*, and *Utf1*, were chosen for validation. QPCR results were in agreement with the sequencing data; all three genes were significantly up-regulated in Fresh-ET (vs. NC) but down-regulated in FET (vs. Fresh-ET), with no significant difference between FET and NC (Fig. 5c). Previous studies have reported a correlation between fetal weight loss and elevated *Cxcl14* expression^(39)^. In summary, speculated that this regression might be underlying the fetal weight improvement in FET.

### Embryo cryopreservation introduced new changes to gene expression compared to Fresh-ET

**Fig. 6:**
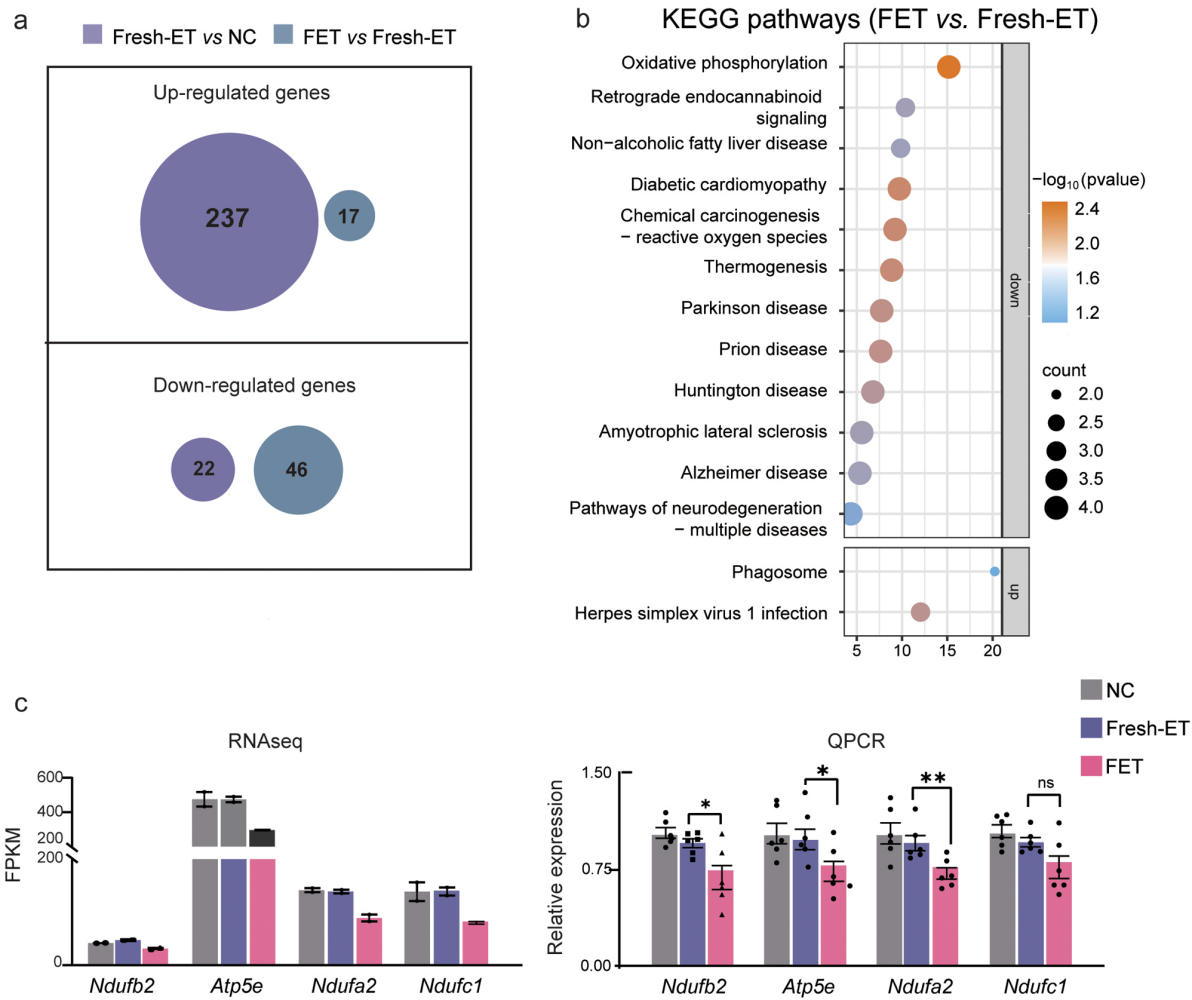
Newly introduced changes by embryo cryopreservation. (**a**) The overlap of up-regulated genes and down-regulated genes between FET vs. NC and FET vs. Fresh-ET. (**b**) KEGG pathway analysis of DEGs of FET vs. Fresh-ET. (**c**) The expression level of four representative genes related to OXPHOS pathway (*Ndufb2, Atp5e, Ndufa2*, and *Ndufc1*). Data came from RNA-seq (upper graph) and QPCR (lower graph). *P < 0.05, **P < 0.01

The regression trend exhibited by FET on both transcriptional level and individual phenotype seemingly implied that it was a beneficial treatment strategy compared to the Fresh-ET, whereas further analysis denied this inference. To determine the independent effects caused by embryo cryopreservation, we specifically focused on DEGs of FET vs. Fresh-ET. A total of 63 DEGs were identified, with 17 genes up-regulated and 46 genes down-regulated in the FET group compared to the Fresh-ET group (Fig. 3a). Notably, we found that the up-regulated genes and down-regulated genes of Fresh-ET vs. NC and FET vs. Fresh-ET did not overlap at all, suggesting a distinct impact induced by embryo preservation compared to Fresh-ET (Fig. 6a). KEGG enrichment analysis showed that the down-regulated genes in FET were involved in oxidative phosphorylation, retrograde endocannabinoid signaling, non-alcoholic fatty liver disease, diabetic cardiomyopathy, chemical carcinogenesis, thermogenesis, Parkinson’s disease, Prion disease, Huntington’s disease, Amyotrophic lateral sclerosis, and Alzheimer’s disease. Up-regulated DEGs were related to phagosome and herpes simplex virus 1 infection (Fig. 6b). Of particular interest, Parkinson’s disease, Prion disease, Huntington’s disease, Amyotrophic lateral sclerosis, and Alzheimer’s disease are all neurodegenerative diseases, which may suggest a profound effect of FET on distant neurological conditions. The oxidative phosphorylation pathway was the most significantly enriched in down-regulated DEGs, QPCR was performed to validate the alteration of the four genes related to this pathway (*Ndufb2*, *Atp5e*, *Ndufa2*, and *Ndufc1*). Experimental results largely confirmed the sequencing data. The expression levels of *Ndufb2, Atp5e, Ndufa2, and Ndufc1* were comparable between Fresh-ET and NC. The former three genes were significantly reduced in FET compared to Fresh-ET, and *Ndufc1* was decreased in FET, whereas there was no statistical significance (Fig. 6c). In conclusion, we inferred embryo cryopreservation as a potentially risky manipulation considering these newly introduced alterations in FET.

### Imprinted genes were abnormally expressed in both Fresh-ET and FET placenta

**Fig. 7:**
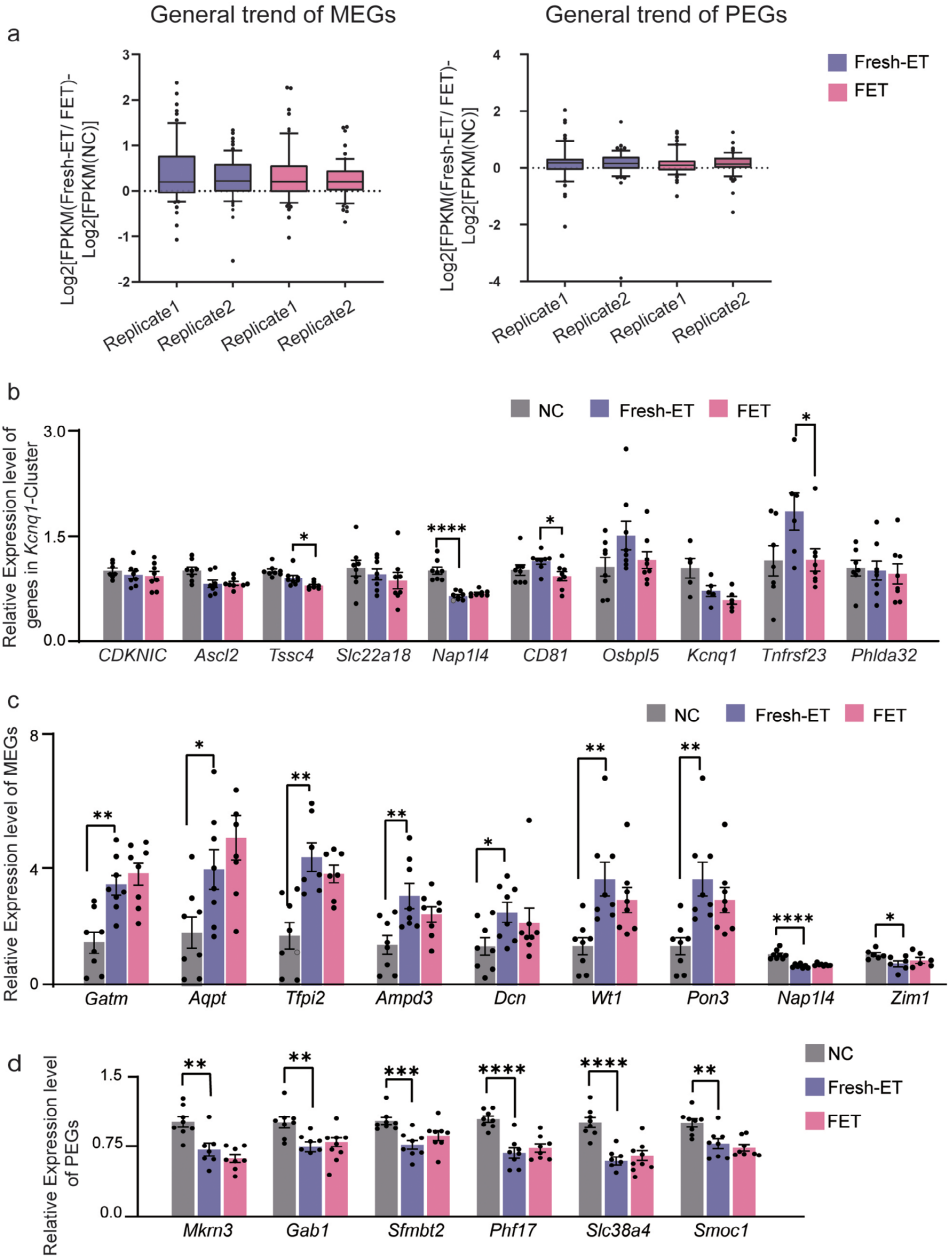
Interrogations of imprinting gene-status in both fresh-ET and FET placenta. (**a**) The overall changing trend of MEGs (left) and PEGs (right) in Fresh-ET and FET groups. The boxplots represent the expression differences of genes compared to the NC group in Fresh-ET and FET groups, calculated as Log_2_[FPKM (Fresh-ET/ FET)- Log_2_[FPKM(NC)]. The replicate 1 refers to Log_2_[FPKM (Fresh-ET 1/ FET 1)- Log_2_[FPKM (NC 1)]; The replicate 2 refers to Log2[FPKM (Fresh-ET 2/ FET 2)- Log2[FPKM (NC 2)]. (**b**) Results of QPCR of all imprinted genes enclosed in Kcnq1-Cluster. (**c**) Results of QPCR of all MEGs that were altered in Fresh-ET compared to NC. (**d**) Results of QPCR of all PEGs that were altered in Fresh-ET compared to NC. MEG, maternal expressed genes; PEG, paternal expressed genes. *P < 0.05, **P < 0.01, ***P < 0.001, ****p < 0.0001

Imprinted genes play a crucial role in the development of placenta and fetus [40]. Figure 7a demonstrated the overall changing trend of all imprinting genes in Fresh-ET and FET groups, classified as maternal expressed genes (MEGs) and paternal expressed genes (PEGs), respectively. The boxplots in these two graphs represent the expression differences of genes compared to the NC group in Fresh-ET and FET groups. In the graph illustrating MEGs, we observed that the expression differences of MEGs in Fresh-ET and FET were largely above zero. This implies that MEGs may have been up-regulated in both Fresh-ET and FET groups compared to the NC group. Furthermore, this up-regulation tendency appeared to be consistent in both Fresh-ET and FET groups, indicating that the changes of MEGs in FET might have been inherited from Fresh-ET. As for PEGs, boxplots demonstrated no significant up- or down-regulation change in either Fresh-ET or FET groups compared to the NC group. Then, we conducted QPCR to explore the expression levels of some specific imprinted genes that might have been altered suggested by sequencing data. Most imprinted genes reside in clusters. As long as there was one significantly altered imprinted gene in a cluster, we verified the expression level of all imprinted genes in the cluster. In addition, the expression level was also validated if imprinted genes from the same parental side within a cluster exhibited a consistent changing trend, even if none of these genes were statistically significantly altered. QPCR results revealed that three protein-coding imprinted genes in the *kcnq1*-cluster were altered in FET, *Tssc4*, *CD81*, and *Tnfrsf23*. These three genes remained unchanged in the Fresh-ET (vs. NC), whereas they were down-regulated in FET (vs. Fresh-ET) (Fig. 7b). As for the comparison between Fresh-ET and NC, a greater number of imprinted genes were altered. QPCR results showed that seven MEGs, *Gtam, Aqpt, Tfpi2, Ampd3, Dcn, Wt1*, and *Pon3*, were up-regulated in Fresh-ET (vs. NC), and two MEGs, *Nap1l4*, and *Zim1* were down-regulated (Fig. 7c). Six PEGs, *Markn3, Gab1, Sfmbt2, Phf17, Slc38a4*, and *smoc1*, were down-regulated in Fresh-ET (vs. NC) (Fig. 7d). All 15 genes that underwent expression changes in Fresh-ET (vs. NC) were not significantly altered in FET compared to Fresh-ET. The above results suggested that both Fresh-ET and FET disrupted the normal expression of imprinted genes, especially MEGs, and the impact of FET might be considerably inherited from Fresh-ET.

## Discussion

This study reported the differential gene expressions among placentas from NC group, Fresh-ET group and FET group. The safety of embryo cryopreservation has been discussed in many articles before. Several papers have investigated the gene expression changes between frozen-thawed embryos and fresh embryos [41–48]. In addition, Yuan Zhu et al. have conducted transcriptome sequencing of E18.5 placenta following Fresh-ET and FET [48]. While the previous articles focused only on the comparison between fresh and frozen groups, we set up a naturally conceived group as a reference under normal physiological conditions. The three groups, NC group, Fresh-ET group, FET group, respectively correspond to three common pregnancy scenarios in clinical practice. We interrogated the expression alteration of genes in the three groups of mid-term placentas and jointly analyzed the DEGs from different comparisons (Fresh-ET vs. NC, FET vs. NC, FET vs. Fresh) to better understand the effects of embryo cryopreservation as an independent influencing factor on placental development, and to compared its effects with traditional Fresh-ET.

It is found that DEGs of FET vs. NC were not equal to the compilations of DEG of FET vs. Fresh-ET and Fresh-ET vs. NC. Therefore, we infer that the influence of embryo cryopreservation manipulation can not be simply taken as an extra accumulation effect adding to the Fresh-ET outcome. All these manipulations involved in the assisted reproduction process disrupt gene expression in a synergetic way and the final consequences are attributed to the whole strategy. A previous study also reported an increase in fetal weight in FET compared to Fresh-ET, which was consistent with our experimental results. By analyzing RNA-seq data, it figured that the *Lncenc1-*miRNA*-Gjb5* axis may be the mechanism underlying the observed weight increase [48]. However, in the present study, the expression of *Gjb5* was comparable in Fresh-ET and FET groups. This discrepancy might be related to the different time points we selected for sequencing.

A previous study conducted on a rabbit model examining the metabolomics of placentas in NC, Fresh-ET, and FET suggested that embryo cryopreservation induced fewer changes compared to other in vitro manipulations of ART, which is consistent with our findings [49]. Nonetheless, it is important to note that embryo cryopreservation still triggered some significant alterations that are absent in Fresh-ET. Specifically, our analysis identified 63 DEGs between FET and Fresh-ET groups. These genes were particularly enriched on oxidative phosphorylation (OXPHOS) pathway and pathways associated with multiple neurodegenerative diseases. There is currently a lack of evidence supporting the relationship between FET and neurodegenerative diseases. This may be because these diseases usually occur late in life, while the application of FET technology has only been promoted in the past decades. Nevertheless, based on the findings of this study, we believe it is essential to track the long-term outcomes and the potential risks of neural diseases of FET offspring. OXPHOS pathway plays a vital role in ensuring the proper functioning of mitochondria and supplying energy to cells and organisms. Upon comparing previous animal experiments related to embryo cryopreservation, we found that the DEGs between frozen and fresh embryos in many of the experiments were also enriched on the OXPHOS pathway [44, 50, 51]. It is reported that impaired OXPHOS would lead to overloaded oxidative stress and excessive ROS [52]. Studies attempting to mitigate the detrimental effects of ART manipulations on embryonic development found that the addition of antioxidants could remarkably improve blastocyst formation rates and their quality [53, 54]. Based on the above findings, we speculate that the deficiency of OXPHOS may underlie the reduced formation rates and quality observed in our experiment following embryo cryopreservation. Furthermore, genes related to the OXPHOS pathway, such as the four genes we observed to be altered in our study (*Ndufb2, Atp5e, Ndufa2*, and *Ndufc1*), could potentially serve as markers for assessing the damage induced by embryo cryopreservation.

Our analysis of the DEGs between Fresh-ET vs. NC and FET vs. NC revealed that they were largely enriched onto the same pathways, such as cell cycle, progesterone-mediated oocyte maturation, motor proteins, p53 signaling pathway, oocyte meiosis, cellular senescence, ECM-receptor interaction, and Cushing syndrome. This finding highlights a significant overlap in genes affected by Fresh-ET and FET, indicating that a substantial portion of the changes observed in FET can be attributed to Fresh-ET process. Moreover, it strongly suggests that the in vitro operations involved in Fresh-ET have a profound influence on genes associated with these pathways. Although our study primarily focuses on the safety of embryo cryopreservation, further exploration of these alterations induced by Fresh-ET is warranted.

The reversal trend of some genes in FET is an interesting finding. Specifically, some genes that were up-regulated in Fresh-ET (vs. NC) showed a downward trend in FET, while genes that were down-regulated in Fresh-ET (vs. NC) demonstrated an upward trend in FET. To the best of our knowledge, no previous study conducted on animal models has reported such phenomena. However, a comparative study based on human placenta and umbilical cord blood did report a similar finding [55]. It was found that the DNA methylation level of the imprinting control region of *H19*-Cluster and transposable element LINE1 was significantly reduced in Fresh-ET compared to NC, while no difference was observed between FET and NC. It was suggested that this unexpected revert might be attributed to the varying hormonal strategies applied on Fresh-ET and FET mothers. However, this speculation can not explain the retrogression observed in our experiments, as the hormonal cycles adopted here were similar between Fresh-ET and FET groups. We hypothesize that the additional manipulations in FET strategy and the relatively lower blastocyst formation rate may have inadvertently eliminated some poor-quality embryos, thus resulting in a greater chance of transferring higher-quality embryos under such unintentional “second selection”. And this “second-selection” may be the origin of the reversal trend of some genes in FET. Among all genes showing a tendency to regress, we focused on *Utf1* and *Cxcl14*, both of which were further validated by QPCR and demonstrated a significant increase in Fresh-ET meanwhile obviously declined in FET. An elevation of *Cxcl14* in the umbilical cord blood of SGA fetuses has been reported [39]. Hence, we infer that the increase of *Cxcl14* in Fresh-ET might be related to its lower fetal weight (vs. NC), whereas the regression in FET could possibly explain the higher fetal weight (vs. Fresh-ET). Moreover, *Utf1* is a predicted upstream transcription factor for *Cxcl14*. Thus, the *utf1-cxcl14* axis may underlie the mechanisms of fetal weight change in Fresh-ET and FET.

Imprinted genes have been attracting extensive attention in ART safety studies. Given the established effects of Fresh-ET and FET on fetal weight and the crucial role of imprinted genes in placenta function, many articles suggest a causal link between the changes of imprinted genes and the impairment of fetal development in ART [56–58]. Consistent with this hypothesis, substantial evidence has supported the alteration of imprinted genes triggered by ART manipulation. Our previous study identified the disruption of three imprinted genes, *PEG10, L3MBTL*, and *PHLDA2*, in ART-conceived children [59]. Additionally, some other studies have reported the alteration of imprinted genes by either embryo cryopreservation or FET, such as *Grb10, H19*, miR-149-5p, *miR-130a-3p*, *miR-487b-3p*, and *miR-423-5p* etc [60–62]. . In this study, we examined all protein-coding imprinted genes in both Fresh-ET and FET, and found only three genes altered in FET group when compared to Fresh-ET group, which were *tssc4*, *CD81*, and *Tnfrsf23*. This finding is not entirely in line with previous articles, and we speculate that the discrepancy may be attributed to differences in protocols and developmental stages. In Fresh-ET group, more variations of imprinted genes were observed. QPCR results showed that, 9 MEGs and 6 PEGs were dysregulated in Fresh-ET (vs. NC). According to the “parental conflict” theory, MEGs tend to restrain excessive maternal nutrition exploitation, whereas PEGs favor unlimited resource acquisition. Thus, the up-regulation of MEGs and down-regulation of PEGs might be underlying the mechanisms of the decreased fetal weight in Fresh-ET observed in our study. Besides, all these fifteen genes that were altered in Fresh-ET showed no expression variations in FET (vs. Fresh-ET). This suggested that FET largely inherited the changes of imprinting genes caused by Fresh-ET.

## Conclusion

In conclusion, our study revealed impaired embryonic developmental potential both before and after implantation following embryo cryopreservation. Whereas, FET somehow ameliorated the restriction of intrauterine fetal growth resulting from Fresh-ET. Changes introduced by embryo cryopreservation were relatively moderate on overall gene expression compared to other in vitro manipulations involved in Fresh-ET. Nonetheless, these changes should not be neglected, especially the alterations related to the OXPHOS pathway, which have been consistently reported in many previous studies as well as this study. Additionally, we unexpectedly noticed some Fresh-ET-altered genes to be restored in FET, and this may help explain the improvement of FET on some Fresh-ET induced perinatal complications. In regard to imprinted genes, we found three genes altered in FET and fifteen genes altered in Fresh-ET. This finding suggests a more significant effect of Fresh-ET on imprinting gene expressions, which should encourage further exploration of imprinted genes in the context of Fresh-ET. Considering the results of our experiments, we deem embryo cryopreservation to be a potentially risky intervention in ART strategy, even though its effects may be less pronounced than those of Fresh-ET.

### Electronic supplementary material

Below is the link to the electronic supplementary material.

Supplementary Material 1

## Data Availability

Sequence data that support the findings of this study are available upon request to the corresponding author.

## Abbreviations

FET: frozen embryo transfer
Fresh-ET: fresh embryo transfer
NC: naturally conceived
IVF: in vitro fertilization
IVC: in vitro culture
HCG: human chorionic gonadotrophin
PMSG: pregnant mare serum gonadotropin
DEG: differentially expressed gene
ART: assisted reproductive technology
SGA: small for gestational age
MEG: maternal expressed gene
PEG: paternal expressed gene
OXPHOS: oxidative phosphorylation
GO: gene ontology
KEGG: kyoto encyclopedia of genes and genomes
